# Acceptability Study of A3-K3 Robotic Architecture for a Neurorobotics Painting

**DOI:** 10.3389/fnbot.2018.00081

**Published:** 2019-01-10

**Authors:** Salvatore Tramonte, Rosario Sorbello, Christopher Guger, Antonio Chella

**Affiliations:** ^1^RoboticsLab, Department of Industrial and Digital Innovation (DIID), Universitá di Palermo, Palermo, Italy; ^2^G.tec Medical Engineering GmbH, Schiedlberg, Austria; ^3^ICAR-CNR, Palermo, Italy

**Keywords:** robot, event related potential (ERP), brain computer interface (BCI), art, human-robot interaction (HRI)

## Abstract

In this paper, authors present a novel architecture for controlling an industrial robot via Brain Computer Interface. The robot used is a Series 2000 KR 210-2. The robotic arm was fitted with DI drawing devices that clamp, hold and manipulate various artistic media like brushes, pencils, pens. User selected a high-level task, for instance a shape or movement, using a human machine interface and the translation in robot movement was entirely demanded to the Robot Control Architecture defining a plan to accomplish user's task. The architecture was composed by a Human Machine Interface based on P300 Brain Computer Interface and a robotic architecture composed by a deliberative layer and a reactive layer to translate user's high-level command in a stream of movement for robots joints. To create a real-case scenario, the architecture was presented at Ars Electronica Festival, where the A3-K3 architecture has been used for painting. Visitors completed a survey to address 4 self-assessed different dimensions related to human-robot interaction: the technology knowledge, the personal attitude, the innovativeness and the satisfaction. The obtained results have led to further exploring the border of human-robot interaction, highlighting the possibilities of human expression in the interaction process with a machine to create art.

## 1. Introduction

### 1.1. Motivation of the Study

The Brain Computer Interface (BCI) is a direct method of communication between a human brain and a computer. It measures brain activity associated with the user's intention and translates the recorded brain activity into corresponding control signals for BCI applications (Graimann et al., 2010). Using BCIs by people with severe paralysis (e.g., Amyotrophic lateral sclerosis (ALS) neurological disease) for communication controlling external devices (Spataro et al., 2017) and for extending the physical presence (Chella et al., 2009), especially in clinical applications, is well known. In the last years BCI technology started to be used to create or modify art-pieces. Users can compose art in real time using brain signals with different paradigms. In Sorbello et al. (2018), a Geminoid (Sakamoto et al., 2007) is used as feedback during music listening to express user's mental states as movement of the Geminoid robot. In Todd et al. (2012) the possibility of using BCI for creative expression as part of an entertainment software package is explained. Münßinger et al. (2010) evaluated the results of a painting application based on brain computer interface on healthy subject and ALS patient. Manipulators are effective devices used mainly in for industrial, manufacturing and medical purposes. KUKA manipulator robot used during the experiment could be programmed using two general methods: manual programming systems and automatic programming system with a BASIC-like syntax and simple commands allocated (Biggs and MacDonald, 2003). Alternatively, Sanfilippo et al. (2015), implemented a cross-platform client to control Kuka, using its standard kinematics. As stated from Cela-Conde et al. (2004) Art is a creation of the Brain, and if we want to understand users' mind it is necessary to begin from creative process of the mind (Folgieri et al., 2013). Vygotsky (1990) has discriminated two levels in creativity human mental activities: the first is mind left to itself (*natural mental process*); the second is mind equipped with tools and auxiliary means (*cultural mental process*). In this context BCI tools are useful to understand and to evaluate the cognitive brain process triggering creativity (Folgieri et al., 2014).

### 1.2. State of Art and Related Work in the Field

Lucchiari et al. (2016) studied connection between cerebral rythms and creative process. Many studies used Brain Computer Interface to create a neuroprosthetic control systems to stimulates organism to reanimate the arts (Moritz et al., 2008; Ethier et al., 2012; Knudsen et al., 2014). The main limitation of these approaches consists in the use of invasive Brain Computer Interface to achieve devices control. Nijholt and Nam (2015) have addressed challenges in designing BCI applications related to the experience of art. Andujar et al. (2015) propose a definition for artistic brain-computer interfaces (artistic BCI) from a passive BCI perspective in four fields: human-computer interaction, neurophysiology, art, and computing. Wadeson et al. (2015) reviewed the literature on artistic BCIs by classifying four types of user control: *selective control, passive control, direct control and collaborative control*. Botrel et al. (2014) explained a Brain Painting for painting using the event related potentials.

### 1.3. Contribution of the Present Study

In the multimodal interaction research using BCI, human-robot interaction aims at supporting with BCI tool the user's desire to find a new way to better exhibit his artistic feelings. In particular in the proposed paper the goal is to monitoring the mental state of an user in term of creativity in order to modify an artistic environment. On the other hand, in the proposed paper, authors present a novel robotic architecture for using robot as a neuroprosthetic extension of the user through a non-invasive Brain Computer Interface. The entire system is composed by a Human Machine Interface based on P300 Brain Computer Interface and a robotic architecture composed by a deliberative layer and a reactive layer to translate user's high-level command in a stream of movement for robots joints. User can give commands to the robot which, thanks to the deliberative architecture is able to accomplish the user's command properly. In particular, user selects a high-level task, for instance a shape or movement, using a brain machine interface and the translation in robot movement is entirely demanded to the Robot Control Architecture defining a plan to accomplish user's task. According to Vygotsky's theory (Vygotsky, 1990), the proposed BCI Robotic Architecture could represent for an user a *stimulus-means* that helps with its condensed experience a different manifestation of his creativity.

## 2. Material and Methods

### 2.1. The A3-K3 Architecture

**Figure 1:**
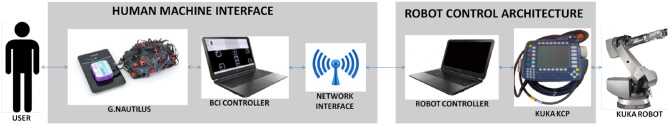
The High level description of the system and of its constituents.

**Figure 2:**
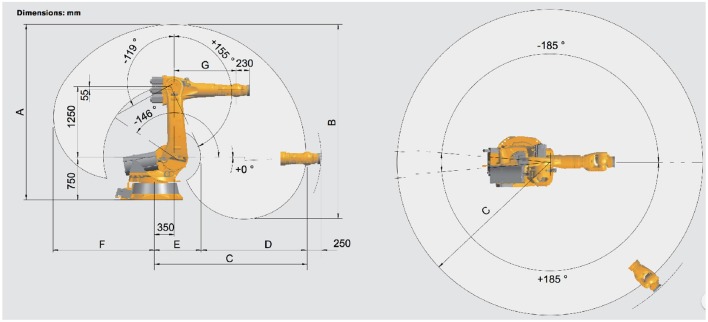
The Kuka Kr 210 -2 robot, The robot has 6 degree of freedom and roto-translations are defined in forward and inverse kinematics. Photo from kuka english brochure pdf file https://www.kuka.com/-/media/kuka-downloads/imported/6b77eecacfe542d3b736af377562ecaa/pf0031_kr_2102_k_en.pdf.

In this section authors first present the architectural concept underling the A3-K3 system and the high-level data flow. Afterwards, a description of the modules that make up the architecture is given. The full architecture, described in Figure 1 provides an high-level vision of the full system. It is composed by two main modules, the Human Machine Interface (*HMI*) and the Robot Control Architecture (*RCA*). The Human Machine Interface is used to give to the user an interface to control the robot. Signals were acquired using an Electroenchephalography (EEG) amplifier and sent to the Brain Controller Interface (BCI) of the Human Machine Interface (HMI). The Brain Controller Module classifies the signals and sends a task over the network system to the Robot Controller Architecture which translated each task in commands for the robot. The robot used is a Series 2000 KR 210-2. The KR 210-2 robot is typically used for industrial applications. It has a total volume of 55 m^3^ and could reach an extension of 2,700 mm. It provides six degrees of freedom and support a payload up to 210 Kg, technical details are reported in Figure 2.

**Figure 3:**
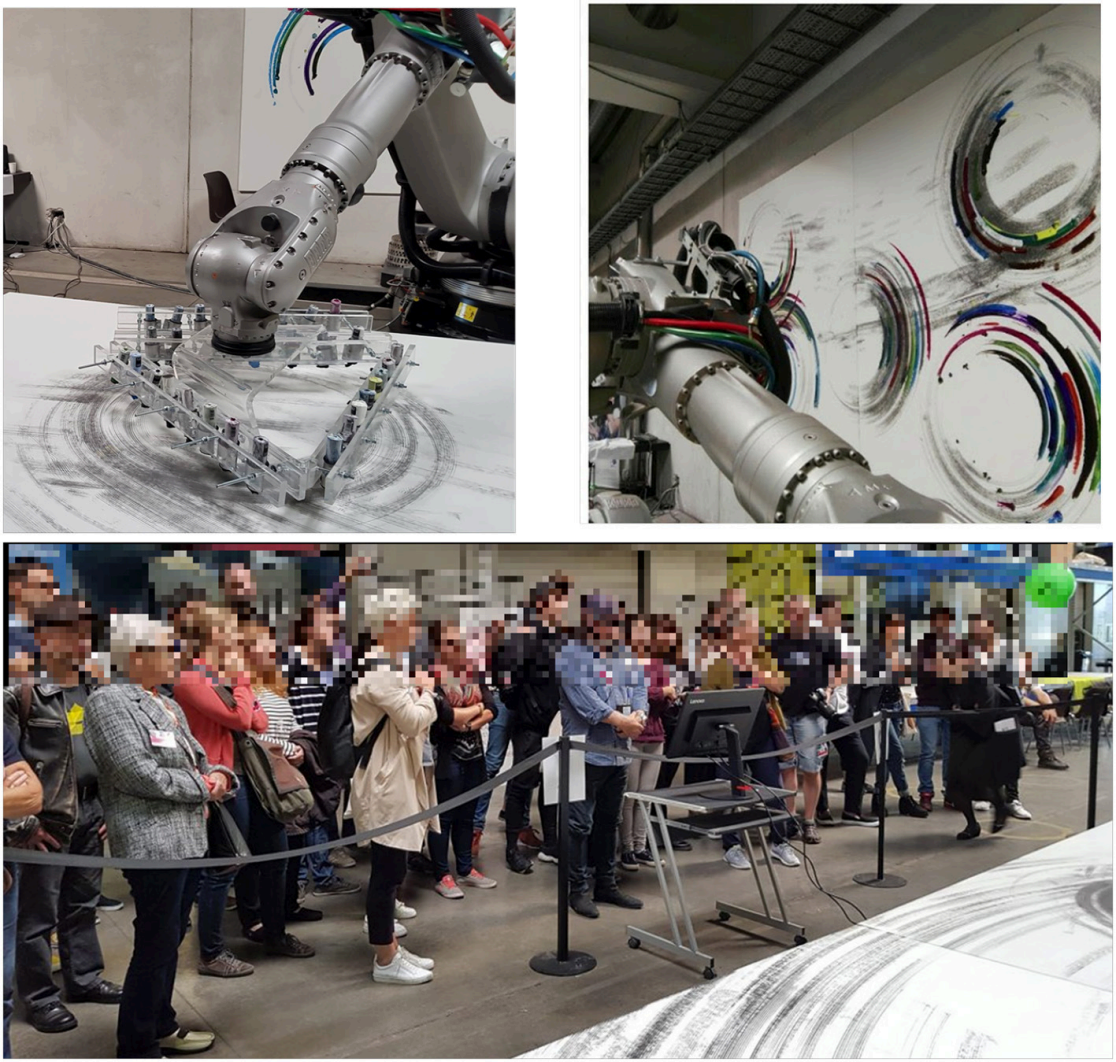
Some images from the experiment conducted during the Ars Electronica Festival.

Robot was equipped with DI Drawing devices that clamp, hold and manipulate various artistic media. For the exhibition pens and pencils have been used, as shown in Figure 3. The presented architecture enables the transmission of high-level tasks to the robot, which execute them calculating the best plan to accomplish user's intention. According to the authors' view, this interaction summarizes the neuroprosthetic concept, since the user can extend his capabilities by controlling a robot with a Brain Computer Interface.

### 2.2. Experimental Procedure and Scenario

The A3-K3 has been first presented at Ars Electronica Festival (Austria) from the 7th to the 11th September 2017. During the festival the system was performed twice per day and a total of ten performances, from now on defined as trials have been held. Each trial lasted 30 minutes and an average of 12 commands per trial were sent to the Kuka robot.

A total of 120 commands has been executed by the robot during the whole exhibition. System was controlled by Dragan Ilic a Serbian artist fascinated by technology and robotics, occasionally visitors had the occasion to test the system but their data has not been taken into account for the present work. To train the HMI a training phase was required. In this phase user was requested to select predetermined symbols from the user interface to train the system over the user's brain response. This procedure required the selection of five symbols for users and lasted approximately 5 minutes. In this phase the Robot Control Architecture provided no feedback. Once the training phase was completed, user tested the correctness of the training phase selecting at least 3 over 5 correct commands. Finally, user was ready to control the robot using the A3-K3 architecture.

**Figure 4:**
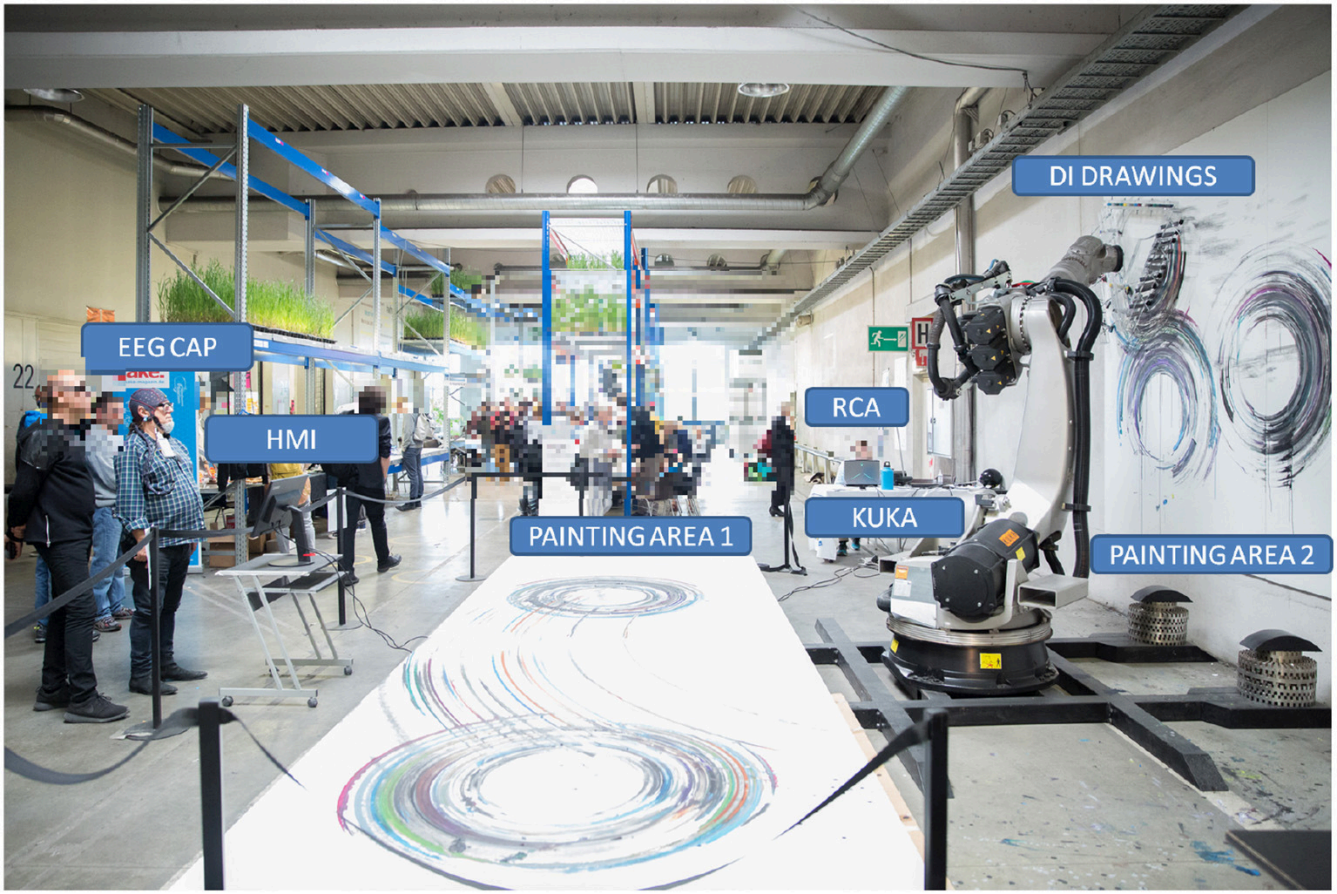
The experimental scenario during the Ars Electronica Festival.

The experimental scenario is described in Figure 4.

User was standing in front of the Kuka robot, wearing an EEG cap. A 22” monitor has been set at 1 meter from the user. The RCA Interface has been implemented on a dedicated laptop. Two painting areas have been set one above the floor, called *Painting area 1* and one above the wall, defined Painting area 2. This experimental study was carried out from all subjects in accordance with the Declaration of Helsinki. All subjects, involved in the experiment, were instructed trough an accurate description of the experiments in English about the phases of the experiment and the goal of the research.

### 2.3. The Human Machine Interface

The Human Machine Interface is the component of the A3-K3 architecture capable of handling human-machine interactions. The interface consists of hardware and software that allow user inputs to be translated as tasks for the Kuka robot. Figure 4 shows the modules that are part of the Human Machine Interface component. The interaction is based on Brain Computer Interface, in particular real time EEG was recorded. The EEG was recorded using the wireless g. Nautilus (gtec, Austria) by the *Data Acquisition module*.Electrodes were set in Fz, Cz, P3, Pz, P4, PO7, Oz, PO8 according to the international 10–20 system, reference left ear mastoid, ground Fpz. The data were collected using a sampling rate of 256 Hz and were transferred in real time via Bluetooth to the receiving PC and bandpass-filtered from 1 to 40 Hz by *the pre-processing module*. A BCI wireless cap has been chosen to give freedom of movement during the performance to the user.

**Figure 5:**
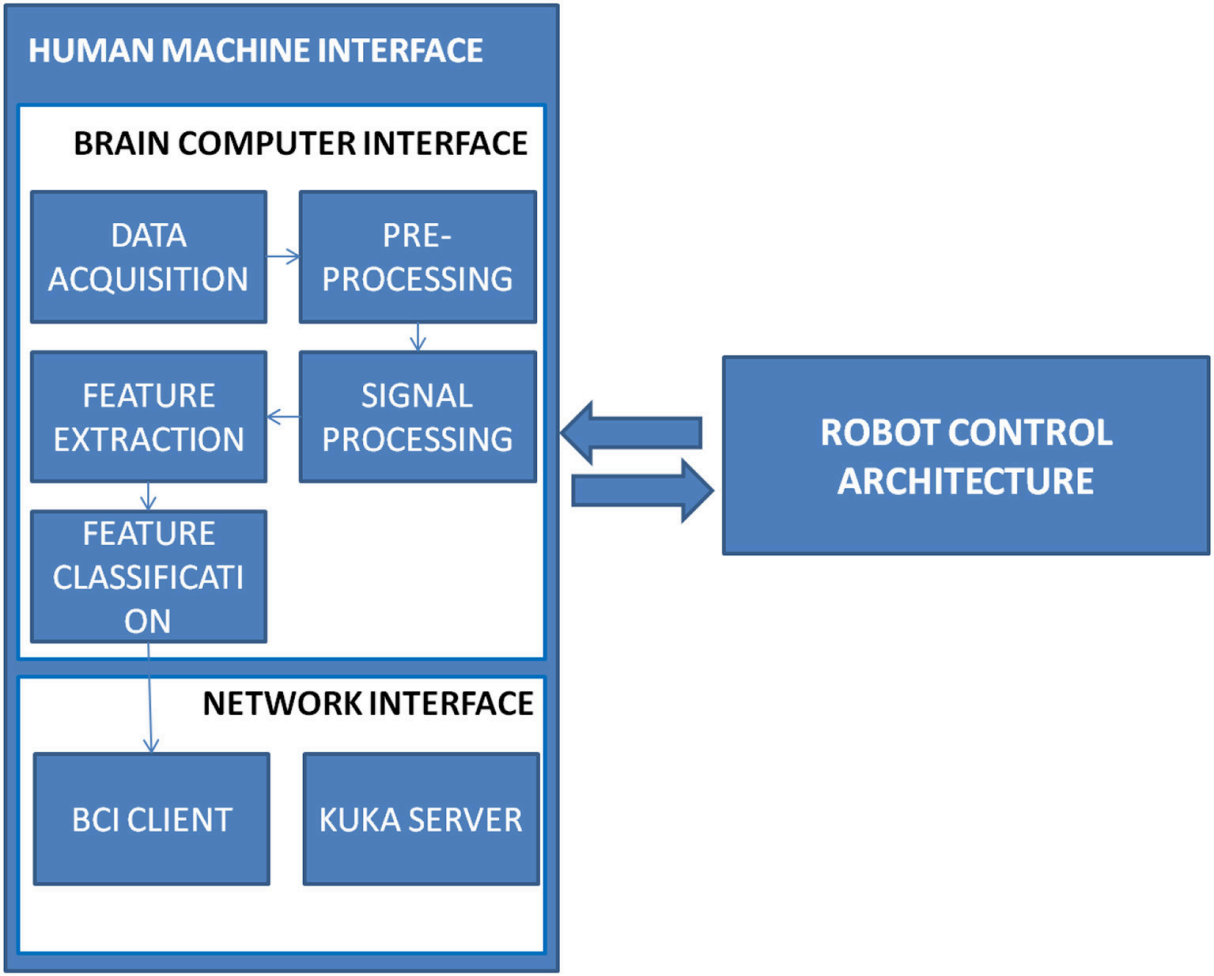
The human machine interaction interface. It is composed by a Brain Computer Interface to interact with the user and by a network module to send commands to the robot controller architecture.

To elicit user's response an ERP interface has been used. The interface chosen is based on the Intendix^TM^ software^1^, a commercial software developed by gtec based on P300 Paradigm. The interface has been customized with icons representing the action that robot will execute. In Figure 5 is shown the user interface (a) and the task associate to each element of the interface (b).

**Figure 6:**
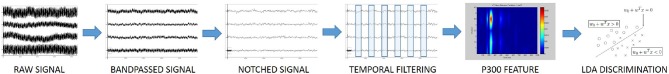
The description of the signal chain.

During each trial, user interface was highlighted using the oddball paradigm. As described in Picton (1992) the oddball paradigm elicits an event-related potentials which occurs in the frontoparietal area of the brain, approximately 300ms after an infrequent stimulus. On the basis of this consideration, frequent and infrequent stimuli are presented to the user, and it is requested to the user to focus on infrequent stimuli, executing a mental process each time an infrequent stimulus occurs. This operation elicits a stronger P300 response (Bennington and Polich, 1999). The *Signal Processing Module* mark the real-time EEG with markers which represent an event (infrequent vs frequent stimulus occurred) to synchronize the EEG with the system event. This step is mandatory for the *feature extraction module* which extract a time-locked window to locate the strongest P300 response. To classify the ERP a one vs. all *latent Dirichlet allocation* (LDA) algorithm (Duda et al., 2012) is applied. The *latent Dirichlet allocation* (LDA) algorithm select one interface item as expected target and considers the other as no-target. In this way the problem to classify the signal is traced back to a two-class problem, lowering the complexity. The process is repeated for each item of the user interface and, at the end the one with the higher response is selected as target and sent to the Network Interface. The signal classification process is described in Figure 6.

### 2.4. The Network Interface

The Network interface (NI) is used to interconnect the Human Machine Interface (HMI) to the Robot Control Architecture (RCA). The Network Interface(NI) is distributed over the HMI and the RCA. In fact the BCI module is implemented inside Intendix Software, while the Kuka Server is implemented on the RCA. Human Machine Interface send an *User Datagram Protocol* (UDP) packet to the BCI Client, which resides on the same machine as the HMI. We decided to implement a *User Datagram Protocol* (UDP) interface because the command is sent just in one packet. The BCI Client is connected to the Kuka server via a *Transmission Control Protocol* (TCP) interface. In this way the Kuka robot can be in a different physical location from the Human Machine Interface (HMI).

### 2.5. The Robot Control Architecture

**Figure 7:**
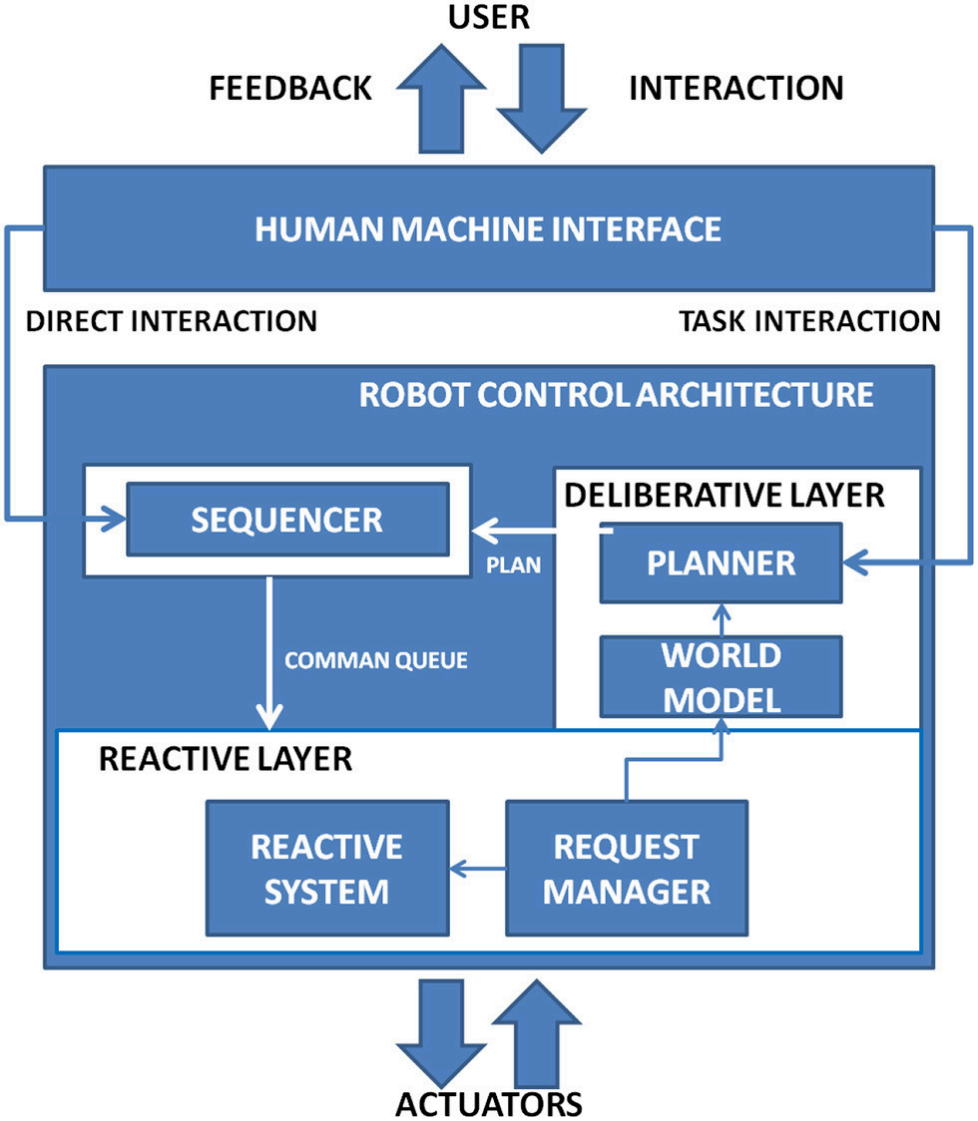
The robotic interface receives commands from the HMI calculates robot state by the symbolic layer and translates command in coordinates and position for the joints of the robot. User can also directly control the robot bypassing the symbolic layer.

The commands received by the Human Machine Interface (HMI) through the Network Interface (NI) is received by the Robot Control Architecture (RCA) as described in Figure 7 and it is based on the model described by Martens et al. (2007).

**Figure 8:**
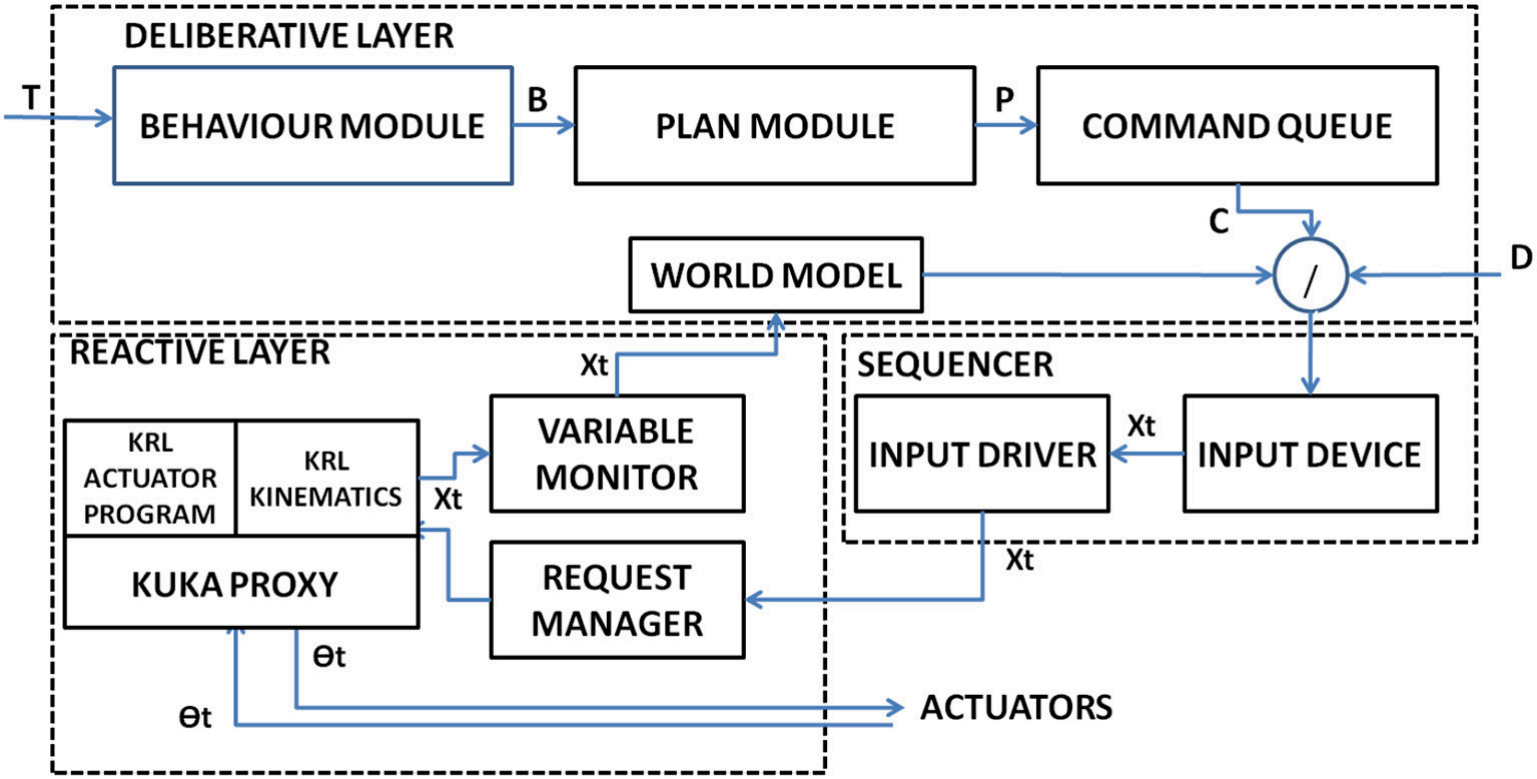
The Insight of the subsystems of the Robot Control Interface.

The Robot Control Architecture (RCA) is a three-level hybrid architecture, which combine a high-level component to define plans with a reactive system to ensure system toughness. The highest level is represented by the *World Model* that is used to define a strategy to execute commands. The *Sequencer* is the middle level and is demanded to the mediation between the plan generated by the world model and the reactive layer. The *Reactive Layer* is directly connected to actuators in a closed loop fashion. Accordingly to the command received two type of interaction are possible: *direct interaction* and *symbolic interaction*. Direct interaction is defined as a direct movement command sent to the robot as direction with a fixed length. *Symbolic Interaction* represents an action required to the robot (e.g., “swipe left over the painting area 1”). In Figure 8 is provided an insight view of the module which constitute the Robot Control Architecture.

#### 2.5.1. The Deliberative Layer

The *deliberative Layer* represents the highest level of abstraction for the robot. It is composed by several modules to translate a high-level command into commands using a planning approach. The Behavior module receives a high-level command, defined Task *T* and produce a behavior *B* where

(1) $$\begin{array}{l} B=\left\{p_{i}:p_{i}\epsilon p_{1}\ldots p_{k}withk<n\right\} \end{array}$$

B is composed by a set *k* of possible *n* plans P where

(2) $$\begin{array}{l} P=\left\{c_{i}:c_{i}\epsilon c_{1}\ldots c_{k}withk<n\right\} \end{array}$$

**Figure 9:**
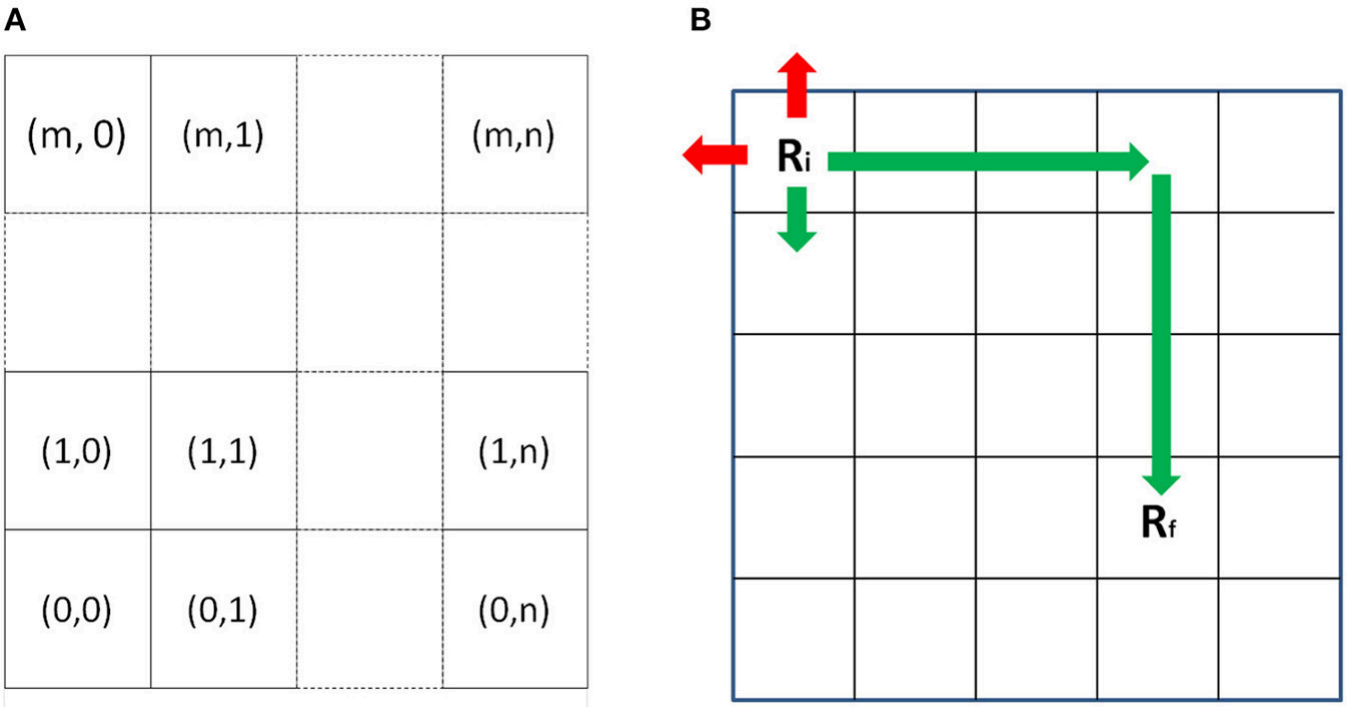
**(A)** The World is represented as a m x n grid. Robot occupies a position on the grid. **(B)** When a command or a direct control is given to the robot, it evaluates if it is possible to accomplish it or not accordingly to his world coordinates.

The emergent plan *P* is obtained from the *planner* and it is decomposed into appropriate “primitive” commands *Cϵc*_*i*_ by the *Command Queue module* to extract the most appropriate *c*_*k*_ from a commands' library tailored to the experiment which constitute the basic building block for robotics actions. To verify if the plan is executable a binary function is defined as If the direct control modality has been set the task is received directly from the HMI and no plan is created. The World model represents the robot position in the environment. As described in Figure 9A, the environment is represented as an *mxn* grid, and robot occupies a grid cell.

As described in Figure 9B, when a new command C or a direct control D is received, it is evaluated the current robot position *R*_*i*_ and the destination position *R*_*f*_ using the following function:

(3) $$\begin{array}{l} F\left(C,W\right)=\{\begin{array}{cc} C & ifW=1\\ 0 & otherwise \end{array} \end{array}$$

if the action is permitted (in green), the command is transferred to the sequencer, otherwise no action is performed (in red).

#### 2.5.2. The Sequencer

The sequencer is the module dedicated to produce the low-level joints motion for the robot actuators. The *Input Device Module* receives the command queue C producing an output vector which is transferred to the *Input Driver Module* connected to the *Request Manager* module which implements a TCP connection.

#### 2.5.3. The Reactive Layer

The reactive layer has been implemented over the JOpenShowVar Architecture, an open-source cross-platform communication interface, developed by Sanfilippo et al. (2015). The input driver sends the command stream to the request manager, connected with the Kuka controller via Transmission Control Protocol/Internet Protocol (TCP/IP). It acts as a middle ware between the network interface and the KRL.

Since it's not possible to directly set manipulator velocity, using KRC cinematic, the target position is calculated as:

(4) $$\begin{array}{l} x_{t}=x_{c}+\overset{t}{\underset{i=0}{\sum }}x_{i} \end{array}$$

where *x*_*t*_ is the target position, *x*_*c*_ is the current position, *x*_*i*_ is the i-th robot position with i varying between 0 and the expected (time) *t* to reach the *x*_*t*_ final position. The actuators translate *x*_*t*_ a vector containing joint configuration accordingly standard KRL kinematics in the Kuka Proxy.

Actuators states are sent to the variable monitor, which is used to derive current robot position of the robot *x*_*t*_.

## 3. Results

To evaluate the general attitude toward the A3-K3 architecture and to understand if people perceived such concept acceptable or not, authors prepared a questionnaire which has been submitted to Ars Electronica Festival visitors^2^ to assess 4 principal dimensions: technology knowledge, attitude, interaction and satisfaction.

The details of people who completed the survey are reported in Table 1.

**Table 1:** Details of people who completed the survey in terms of age, provenance and sex.

Age	18+	18–29	30–39	40–49	50–59	60–69	70+
	69	147	228	97	49	48	39
Provenance	America	Africa	Asia	Europe	Oceania		
	19.38%	5.14%	12.19%	57.12%	6.17%		
Sex	Male	Female	Prefer not to say				
	401	245	681				

A total of 681 people (401 Male, 245 Female, 35 N.A), coming from 4 continents (America 19.38%, Africa 5.14%, Asia 12.13%, Europe 57.12%, Oceania 6.17 %) completed the survey. The most representative group age was 30–39 years (33.48%) and 18–29 years (21.59%).

The questionnaire was composed by the following 3-point Likert questions:

1. Did you know the Kuka robot?
2. Did you know the Brain Computer Interface?
3. Do you think robots can be used to create art?
4. Do you think the brain computer interface is a useful technology?
5. Was the interaction between the robot and the artist natural?
6. Was the robot an extension of the artist?
7. Was the performance innovative?
8. Did you enjoyed the overall performance?

**Figure 10:**
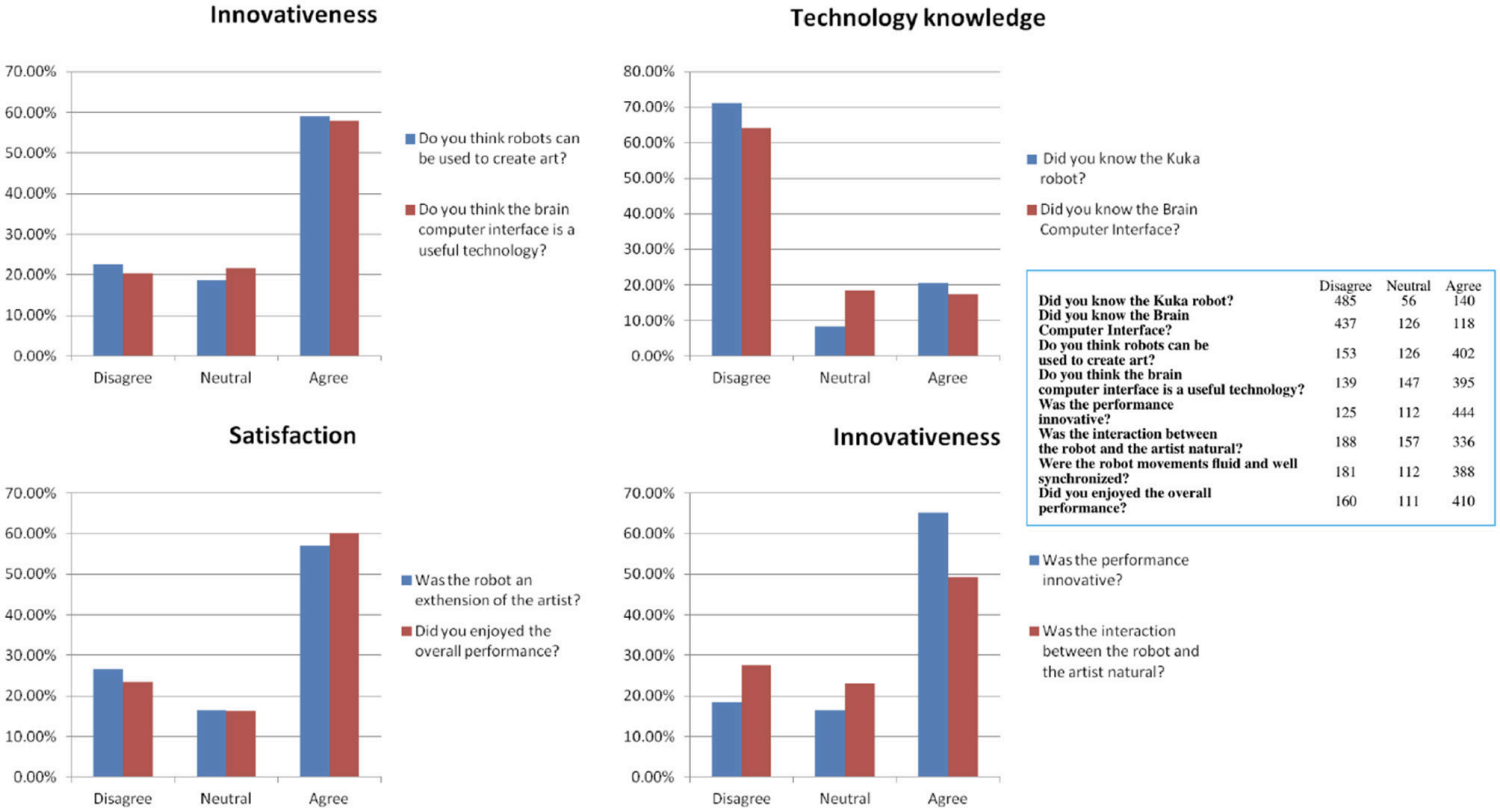
The questionnaire results in terms of 4 dimensions: Technology knowledge, Innovativeness, Personal Attitude and Satisfaction.

Questions 1 and 2 refer to technology knowledge, questions 3 and 4 refers to personal attitude toward Brain Computer Interface and Robotic arts, questions 5 and 6 refers to the perceived quality in the interaction, questions 7 and 8 refers to the satisfaction perceived during the performance. The technology knowledge of BCI and Kuka robots appear to be quite low, in fact only the 20.56% of people had a previous knowledge of the Kuka robot and the 17.33% of people known the Brain Computer Interface. Nevertheless, the personal attitude toward the architecture is well-established, since the 59.03% of people considered possible to use the robot for making art and the 58% of people considered Brain Computer Interface a useful technology. The performance has been considered innovative by the 65.20% of interviewed and the 49.34% of people considered the interaction of the robot with the artist, natural. The robot has been considered an extension of the artist himself by the 56.98% of people and the 60% of people was satisfied by the whole performance. The completeness of the questions and answers given to the survey can be checked in Figure 10 and in Table 2.

**Table 2:** The full list of answer to the survey.

	**Disagree**	**Neutral**	**Agree**
Did you know the Kuka robot?	485	56	140
Did you know the Brain Computer Interface?	437	126	118
Do you think robots can be used to create art?	153	126	402
Do you think the brain computer interface is a useful technology?	139	147	395
Was the performance innovative?	125	112	444
Was the interaction between the robot and the artist natural?	188	157	336
Were the robot movements fluid and well synchronized?	181	112	388
Did you enjoyed the overall performance?	160	111	410

## 4. Discussion

Presented results shown in Figure 10 that industrial robot like Kuka and Brain Computer Interface are still not well known by the majority of people. Nevertheless, people appear to have a positive attitude toward them, accepting them has useful technology in general and in particular to make art. The performance has been considered innovative by the majority of people and only the 18.36% of people didn't appreciate it. The most controversial question is about the spontaneity of interaction between user and robot, since it is not considered “natural” by the majority of people but only by the 49.34% of them. Authors plans to test the system using the low-level command mode, where the users can freely move the Kuka robot on the canvas using BCI, to explore if the modification of the command paradigm, reducing the role of the deliberative layer and giving more freedom of control to the user, will raise the perception of “natural control” in the audience. Interesting to notice that the 56.98% of people considered the robot as an extension of the artist, and they were satisfied by the whole performance.

## 5. Conclusions

In conclusions, authors presented a novel BCI architecture for controlling a robotic arm. The architecture has been designed to be modular with two main systems, the Human Machine Interface and the Robot Control Architecture. Robot architecture implemented a deliberative layer to create a plan to accomplish a high level command selected by the Human Machine Interface. A low-level command modality has been also implemented but not used in presented experiment. The architecture has been tested in Ars Electronica Festival, in September 2017 in Linz Austria and a survey has been submitted to the visitor to address 4 dimensions: technology knowledge, personal attitude, Innovativeness and Satisfaction. Results appear to be promising and the system was well accepted and satisfied people who took part to the survey. Future study will explore how different type of control will change the system perception. In particular authors will change the BCI paradigm, using motor imagery and Steady States Visual Evoked Potentials (SSVEP) and will use the low-level command modality to explore a different Human-Robot Interaction.

## Ethics Statement

All procedures followed were in accordance with the ethical standards of the responsible committee on human experimentation (institutional and national) and with the Helsinki Declaration of 1975, as revised in 2000. Informed consent was obtained from all patients for being included in the study.

## Author Contributions

RS and AC supervised the paper writing and designed the architecture. ST designed and implemented the architecture and conducted the experiments. CG provided hardware and software and reviewed the paper.

### Conflict of Interest Statement

RS, ST, and AC declare that the research was conducted in the absence of any commercial or financial relationships that could be construed as a potential conflict of interest. CG is the CEO of g.tec medical engineering.

## Footnotes

^1^http://www.gtec.at/Products/Complete-Solutions/intendiX-Specs-Features

^2^Please refer to this link to access the questionnaire: https://docs.google.com/forms/d/e/1FAIpQLSfFT6wK7Rlp98qnMe2EcH7VksMiAugqV6SBuDJEDUe97Mc2gw/viewform
